# Anti-breast tumor activity of Eclipta extract *in-vitro* and *in-vivo*: novel evidence of endoplasmic reticulum specific localization of Hsp60 during apoptosis

**DOI:** 10.1038/srep18457

**Published:** 2015-12-17

**Authors:** Rakesh K. Arya, Akhilesh Singh, Navneet K. Yadav, Srikanth H. Cheruvu, Zakir Hossain, Sanjeev Meena, Shrankhla Maheshwari, Anup K. Singh, Uzma Shahab, Chetan Sharma, Kavita Singh, Tadigoppula Narender, Kalyan Mitra, Kamal R. Arya, Rama K. Singh, Jiaur R. Gayen, Dipak Datta

**Affiliations:** 1 Biochemistry Division, CSIR-Central Drug Research Institute (CDRI), Lucknow-226031, India; 2 Toxicology Division, CSIR-CDRI, Lucknow-226031, India; 3 Pharmacokinetics and Metabolism Division, CSIR-CDRI, Lucknow-226031, India; 4 Academy of Scientific and Innovative Research, New Delhi, India; 5 Botany Division, CSIR-CDRI, Lucknow-226031, India; 6 Electron Microscopy Unit, CSIR-CDRI, Lucknow-226031, India; 7 Medicinal and Process Chemistry Division, CSIR-CDRI, Lucknow-226031, India

## Abstract

Major challenges for current therapeutic strategies against breast cancer are associated with drug-induced toxicities. Considering the immense potential of bioactive phytochemicals to deliver non-toxic, efficient anti-cancer therapeutics, we performed bio-guided fractionation of *Eclipta alba* extract and discovered that particularly the chloroform fraction of *Eclipta alba* (CFEA) is selectively inducing cytotoxicity to breast cancer cells over non-tumorigenic breast epithelial cells. Our unbiased mechanistic hunt revealed that CFEA specifically activates the intrinsic apoptotic pathway by disrupting the mitochondrial membrane potential, upregulating Hsp60 and downregulating the expression of anti-apoptotic protein XIAP. By utilizing Hsp60 specific siRNA, we identified a novel pro-apoptotic role of Hsp60 and uncovered that following CFEA treatment, upregulated Hsp60 is localized in the endoplasmic reticulum (ER). To our knowledge, this is the first evidence of ER specific localization of Hsp60 during cancer cell apoptosis. Further, our LC-MS approach identified that luteolin is mainly attributed for its anti-cancer activities. Moreover, oral administration of CFEA not only offers potential anti-breast cancer effects *in-vivo* but also mitigates tumor induced hepato-renal toxicity. Together, our studies offer novel mechanistic insight into the CFEA mediated inhibition of breast cancer and may potentially open up new avenues for further translational research.

Breast cancer is the most commonly diagnosed cancer and the leading cause of cancer related deaths in women worldwide, with an estimated 1.7 million new cases and 522,000 deaths being reported around the world in 2012 alone^1^. Owing to this increased global burden and considering the drawbacks involved in current treatment methods having toxic side effects, search for new and effective therapy is thus of prime importance. Regardless of the recent domination of synthetic chemistry as a method to discover and develop new drugs, the potential of bioactive plant extracts to deliver non toxic, efficient anti-cancer therapeutics is still enormous^2^,^3^,^4^. Historically, natural products have not only offered us new anti-cancer drugs but have also provided unique novel pharmacophores/clues, by which synthetic chemists have synthesized modern day drugs^5^,^6^,^7^.

According to World Health Organization (WHO), 80% people across the globe use medicinal plants for the treatment of several diseases including cancer due to their easy accessibility, cost effectiveness, and less toxic side effects^8^. Medicinal herb *Eclipta alba,* also known as “Bhringraj” in Ayurveda has been widely used as a hepatoprotective agent for ages but the anti-tumor properties of its extract have recently been reported^9^,^10^,^11^. However, the identification of active molecules and their detailed mechanism of action in a particular disease area are lacking. Though flavonoids such as luteolin, apigenin are present in *E. alba,* coumarins like wedelolactone have been reported to be one of the major players of its diverse bioactivities including anti-tumor properties^12^,^13^,^14^. Wedelolactone, luteolin and/or structurally similar apigenin being naturally occurring compounds are generally safe and associated with low toxicity, making them ideal candidates for selectively inducing apoptosis in cancer cells^15^. They generally promote its apoptotic effect via either turning on extrinsic or intrinsic apoptotic pathways or may be both, depending on the cellular context^15^,^16^,^17^.

Intrinsic or mitochondria-mediated apoptotic pathway primarily involves dissipation of the mitochondrial membrane potential and release of apoptogenic proteins into the cytosol, which in turn activates downstream caspases^18^. Therefore, subcellular localization of a certain protein in a particular context dictates the cellular fate for either survival or death. Molecular chaperones like heat shock protein (Hsp) 60 is known to be mainly located in mitochondria and found to be overexpressed in some tumors implying its classical pro-survival function like other Hsps^19^,^20^,^21^,^22^. However, recent reports indicate that Hsp60 could play a dual role in regulating apoptosis either by accumulating at a certain organelle or by being released into the cytoplasm^23^,^24^,^25^,^26^,^27^.

Here, we report that the chloroform fraction of *E. alba* (CFEA) markedly reduce breast tumor growth *in-vitro* and *in-vivo* by selectively regulating the expression of Hsp60 along with altering the other members of intrinsic apoptotic pathway without having any toxic side effects. For the first time, we provide strong evidence that Hsp60 is localized in the endoplasmic reticulum (ER) during breast cancer cell apoptosis. In addition, adapting mass fingerprinting approach, we have also identified that anti-tumor activity of this particular fraction against breast cancer cells is mainly contributed by the presence of flavonoid luteolin instead of the frequently known coumestan wedelolactone.

## Results

### CFEA poses tumor cell selective cytotoxic effects and potentially induces apoptosis in breast cancer cells

**Figure 1:**
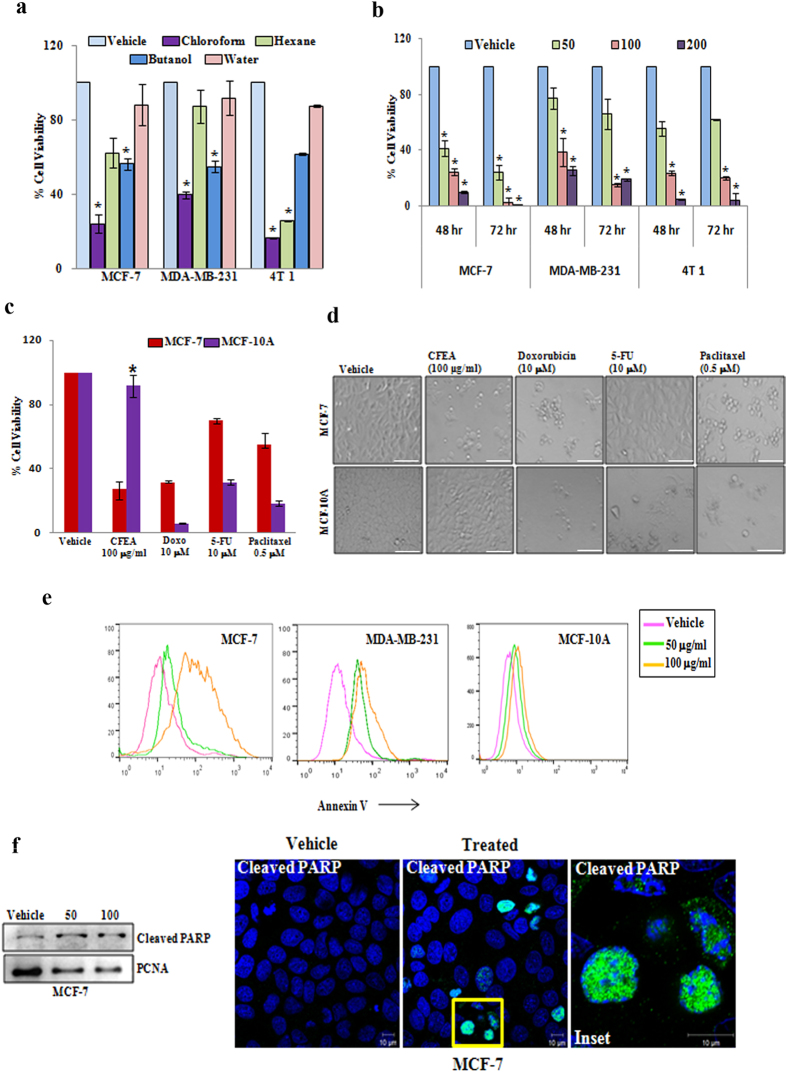
CFEA induces breast cancer cell selective cytotoxic effects and promotes apoptosis. (*a*) MCF-7, MDA-MB-231 and 4T1 cells were treated with 100 μg/ml of different fractions (chloroform, hexane, n-butanol and water) of *E. alba* for 48 hours and cytotoxicity was measured by SRB assay as described in materials and methods. Percent cell viability were tabulated. *Columns*, average of triplicate readings of samples; *error bars*, ± S.D. *p < 0.01, compared to vehicle treated cells. (**b**) MCF-7, MDA-MB-231 and 4T1 cells were treated with increasing concentrations 50, 100, and 200 μg/ml of CFEA for 48 and 72 hours and cytotoxicity was assessed by SRB assay. *Columns*, average of triplicate readings of samples; *error bars*, ± S.D. *p < 0.01, compared to vehicle treated cells. (*c*) MCF-7 (red bar) and MCF 10A (violet bar) cells were treated with CFEA and FDA approved standard anti-cancer drugs Doxorubicin (10 μM or 5.79 μg/ml), Paclitaxel (0.5 μM or 0.42 μg/ml), and 5-Fluorouracil (10 μM or 1.3 μg/ml) for 48 hours and cytotoxicity was assessed by SRB assay. *Columns*, average of quadruplet readings of samples; *error bars*, ± S.D. *p < 0.01, compared to MCF 10A cells treated with different standard drugs. (**d**) Respective photomicrographs of vehicle and treated (48 hours) cells were shown. Scale bar, 100 μm. (**e**) Analysis of apoptosis induced by CFEA in MCF-7 (left panel), MDA-MB-231 (middle panel) and MCF 10A (right panel) cell lines. Cells treated with CFEA for 24 hours were stained with Annexin-V Alexafluor 488 and analysed by flowcytometry. (**f** ) Western blot analysis (left panel) of Cleaved PARP in nuclear fraction of 24 hours post vehicle and CFEA treated (50 and 100 μg/ml) MCF-7 cells. Vehicle or CFEA (100 μg/ml) treated (24 hours) cells were also stained with cleaved PARP antibody and analysed by confocal microscopy (right panel). Scale bar, 10 μm. Results shown from (a) to (**f**) sections are representative of at least three independent experiments.

It has been reported that the extract of *E. alba* has anti-proliferative potential^10^. Here, we separated the methanol extract of *E. alba* into four different fractions–chloroform, n-butanol, hexane, aquatic and investigated the effect of these fractions (100 μg/ml) on *in-vitro* cytotoxicity in three different breast cancer cell lines (non-metastatic human MCF-7, metastatic human MDA-MB-231, and metastatic mouse 4T1) using SRB assay. Out of these fractions, CFEA was found to be the most potent in inducing cytotoxic effect against various breast cancer cells (Fig. 1a) though the aquatic or water fraction did not show any significant cytotoxic effect. Next, we evaluated the dose and time dependent cytotoxic effect of CFEA. As shown in Fig. 1b, 50–200 μg/ml of CFEA dose dependently induced significant cell death in all three breast cancer cell lines compared to control. To test the cancer cell specific cytotoxicity of our fraction in comparison to the marketed breast cancer drugs, we next examined the effect of CFEA and multiple FDA approved cancer drugs (Doxorubicin, Paclitaxel, 5-Fluorouracil) on non-tumorigenic and non-transformed breast epithelial cells MCF 10A versus tumorigenic transformed MCF-7 cells. Most strikingly, we observed (Fig. 1c–d and Supplementary Information Fig. SI 1) that all three cancer drugs were found to be more cytotoxic in MCF 10A cells as compared to MCF-7 cells. In contrast, CFEA has shown minimal cytotoxicity against MCF 10A but on the same dose, it poses marked cytotoxic effects to MCF-7 cells. At least in terms of inducing *in-vitro* cytotoxic effects against breast cancer cells, CFEA poses very good tumor cell selective effects. To validate whether this particular CFEA induces apoptosis in breast cancer cells, we performed Annexin*-*V staining to detect early apoptotic cells and analyzed by flow cytometry. In both MCF-7 and MDA-MB-231 cells, CFEA dose dependently increased early apoptotic cells compared to vehicle treated cells as indicated by the right shift of the histogram overlays, however, a very minimal shift was observed in MCF 10A cells after CFEA treatment indicating CFEA has less cytotoxic effects in non-transformed MCF 10A cells (Fig. 1e). PARP cleavage is a biochemical hallmark of apoptosis and thus to further validate CFEA mediated apoptotic process, we assessed the expression of cleaved PARP in MCF-7 cells by Western blot analysis as well as immunofluorescence staining. For immunoblot analysis, we isolated protein from nuclear fraction of vehicle and CFEA treated cells. As observed by immunoblot and confocal photomicrographs (Fig. 1f) CFEA treatment for 24 hours in MCF-7 cells resulted in significant induction of cleaved PARP expression compared to vehicle treated cells. Thus, together our data suggest that CFEA is able to selectively induce apoptosis in breast cancer cells.

### CFEA selectively promotes intrinsic apoptotic pathways in breast cancer

**Figure 2:**
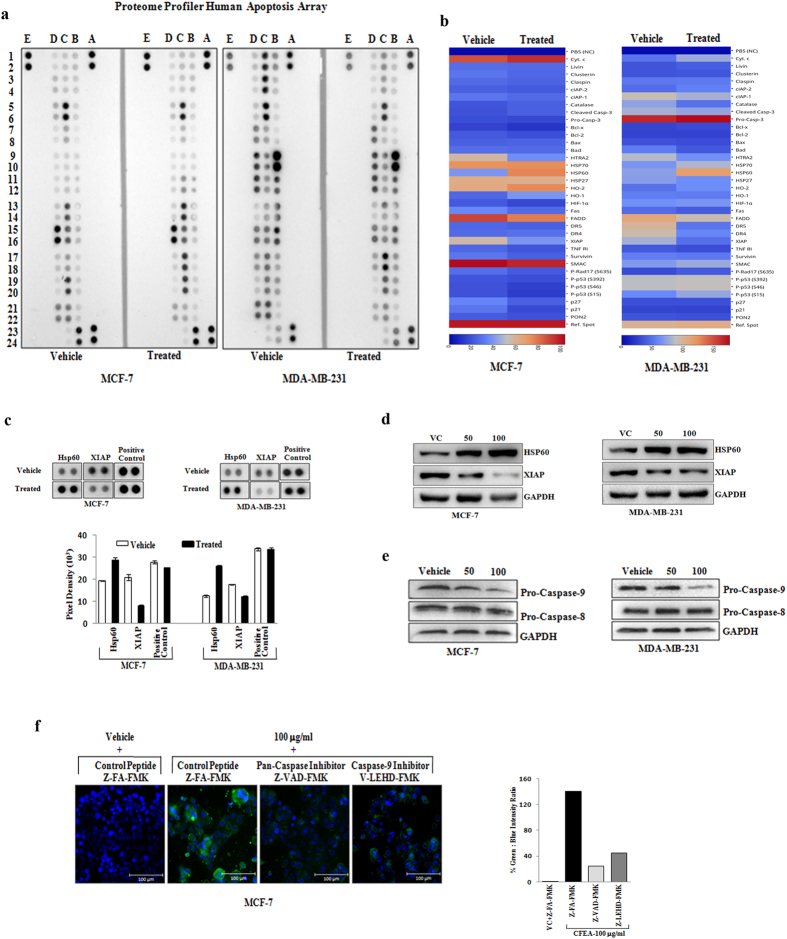
CFEA selectively activates intrinsic apoptotic pathways in breast cancer. (**a–d**) Human breast cancer MCF-7 and MDA-MB-231 cells were treated with CFEA (100 μg/ml) or otherwise mentioned for 24 hours and cells were harvested for protein extraction and analyzed for the expression of apoptotic genes by utilizing proteome profiler apoptosis array or individual western blot analysis. (**a**) Chemiluminescent image of the expression of 35 apoptosis related genes with positive and negative controls in duplicates for vehicle as well as CFEA treated cells were shown. (**b**) Heatmaps depicting differentially regulated proteins in MCF-7 (left) and MDA-MB-231 (right) either after vehicle or CFEA treatment. (**c**) The enlarged images of selected apoptotic proteins found to be markedly altered in the proteome profiler array were shown. Pixel intensity graph (lower panel) of altered target proteins and control spots were displayed. (**d**) Immunoblot images demonstrate individual western blot analysis of Hsp60 and XIAP in vehicle and treated cells. (**e**) Western blot analysis of Pro-Caspase-9 and Pro-Caspase-8 in vehicle and CFEA treated cells. (**f** ) MCF-7 cells were pre-treated with 25 μM of either control peptide or pan-caspase inhibitor Z-VAD-FMK or caspase-9 inhibitor V-LEHD-FMK for 6 hours and then treated with either vehicle or 100 μg/ml of CFEA for 24 hours. Cells were stained with Annexin-V Alexafluor 488 and Hoechst 33342 and analysed under fluorescence microscope. Scale bar, 100 μm. Percentage of green: blue intensity ratio is calculated by NIS Basic Research Software (Nikon) and represented in bar diagram in right hand panel. Representative of three independent experiments.

To explore the molecular mechanism of CFEA induced breast cancer cell apoptosis, we made use of an apoptosis antibody array (ARY009, R&D Systems)^28^. This array platform is unique and an unbiased approach to study the simultaneous expression of 35 apoptosis related proteins spanning both intrinsic and extrinsic pathways. To particularly dissect the CFEA mediated apoptotic signals, MCF-7 and MDA-MB-231 cells were treated with 100 μg/ml of CFEA for 24 hours and antibody array was performed by following the manufacturer’s protocol. Our array blots and heatmaps generated from blots clearly showed that a number of apoptotic signaling proteins were modulated following treatment of CFEA (Fig. 2a–c, Supplementary Information Fig. SI 2). Interestingly, CFEA in both MCF-7 and MDA-MB-231 cells selectively upregulated the expression of Hsp60 compared to control though the other Hsp members like Hsp27 and Hsp70 present in the array remain unaltered. In contrast, marked downregulation of the expression of anti-apoptotic protein XIAP was observed in both MCF-7 and MDA-MB-231 cells (Fig. 2a–c, Supplementary Information Fig. SI 2). Additionally, we performed individual western blot analysis for Hsp60 and XIAP protein expression after two doses (50 and 100 μg/ml) of CFEA treatment. As shown in Fig. 2d, treatment of CFEA resulted in marked upregulation of Hsp60 protein expression and concomitant downregulation of anti-apoptotic protein XIAP in both MCF-7 and MDA-MB-231 cells. Altogether, changes observed after CFEA treatment are particularly selective and indicative for the involvement of the intrinsic apoptotic pathway.

### Inhibition of intrinsic apoptotic pathways diminish cytotoxic effects of CFEA in breast cancer

Loss of mitochondrial membrane potential (MMP) is an integral part of the intrinsic pathway^18^. To confirm the mitochondrial dysfunction in CFEA mediated intrinsic apoptosis, control and treated cells were labelled with fluorochrome JC-1 and analysed by flow cytometry. Representative FACS plots (Supplementary Information Fig. SI 3) for red-FL2-H (indicator of an intact MMP) and green-FL1-H (indicator of loss of MMP) showed that vehicle treated cells mostly exhibit red fluorescence indicating an intact mitochondrial membrane potential. CFEA dose dependently increased the number of cells showing green fluorescence suggesting the disruption of mitochondrial membrane potential. Caspases are the key downstream modulators of any apoptotic process and particularly, activation of caspase-8 and caspase-9 are classical discriminators of the extrinsic and intrinsic pathways of apoptosis respectively^29^. To confirm the selective involvement of intrinsic pathway, we first tested the effect of CFEA on caspase-8 and caspase-9 expression in both MCF-7 and MDA-MB-231 cells by western blot analysis. Here, we observed that CFEA treatment in both the cell lines, preferentially downregulates the expression of pro-caspase-9 compared to vehicle treated cells whereas, pro-caspase-8 expression remains unaltered (Fig. 2e).

We next tested the functional role of caspases and particularly caspase-9 in CFEA induced apoptosis. We assessed the effect of a pan-caspase inhibitor Z-VAD-FMK and caspase-9 inhibitor V-LEHD-FMK and control peptides on CFEA (100 μg/ml) mediated apoptosis of MCF-7 cells by Annexin-V Alexafluor 488 staining. Results showed a marked protection towards CFEA treatment upon exposure to pan-caspase and caspase-9 inhibitors as evident by decreased staining with Annexin-V in treated cells compared to control (Fig. 2f). Therefore, our results put forward that caspases in general as well as selectively caspase-9 inhibition considerably blocked CFEA induced apoptosis in breast cancer cells.

### CFEA mediated upregulation of Hsp60 is pro-apoptotic in function

**Figure 3:**
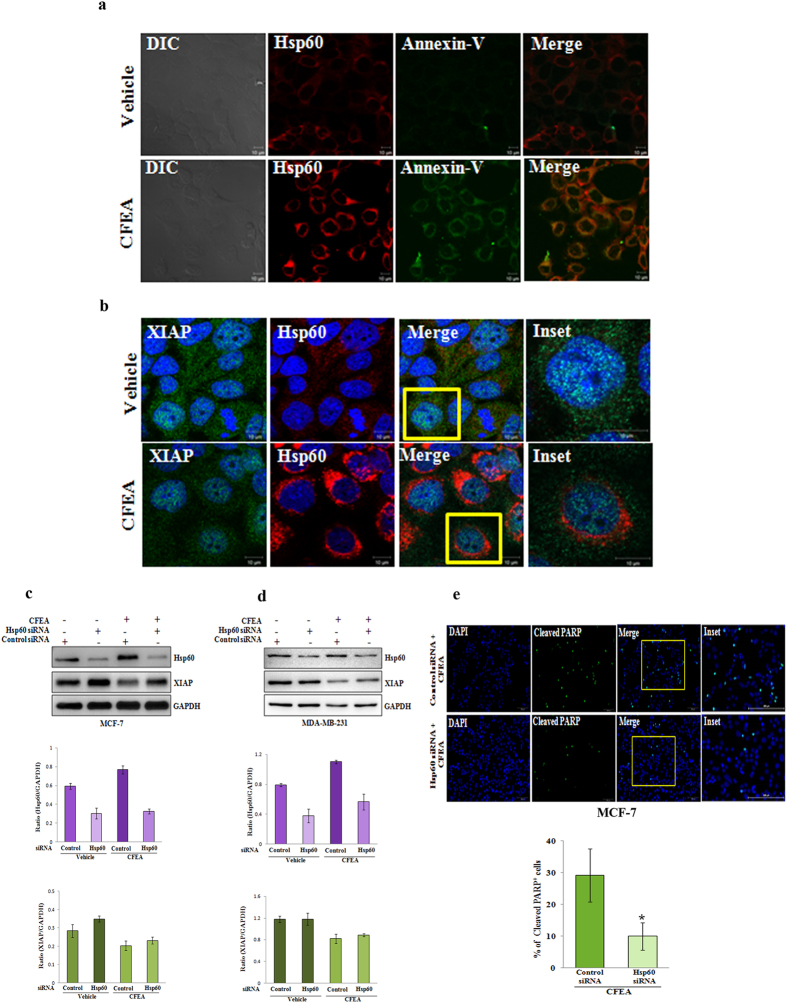
CFEA upregulated Hsp60 is pro-apoptotic in function. (**a–b**) MCF-7 cells were grown in coverslips and treated with either vehicle or CFEA (100 μg/ml) for 24 hours and subjected to immunofluorescence staining and analysed by confocal microscope. Merged confocal pictures represent the superimposition of green and red images and the magnified area of the box where applicable were shown in inset pictures. Representative of three independent experiments. (**a**) Cells were co-stained with Hsp60 (red) and Annexin-V (green) antibodies. (**b**) Cells were co-stained with Hsp60 (red) and XIAP (green) antibodies. Scale bar, 10 μm. In (**c–d**), MCF-7 and MDA-MB-231 cells were transfected with either control or Hsp60 siRNA (50 nM) for 24 hours followed by the treatment with 100 μg/ml of CFEA for another 24 hours, cells were harvested for western blot analysis of Hsp60, XIAP, and GAPDH expression (Top panel); Bar diagrams (bottom panels) show densitometry analysis of HSP60 and XIAP protein expression normalized to GAPDH expression. (**e**) MCF-7 were transfected with either control siRNA or Hsp60 siRNA (50 nM) for 24 hours followed by the treatment with 100 μg/ml of CFEA for another 24 hours, cells were stained with anti-cleaved PARP antibody followed by fluorescent labelling and DAPI staining and visualized under fluorescent microscope in 20 × 10 magnification. Scale bar, 200 μm. In lower panel, % of green cleaved PARP positive cells over total (DAPI stained) number of cells were calculated in 7 different fields of control and siRNA transfected CFEA (100 μg/ml) treated cells and represented in bar diagram; *bars*, +/− SD of control and treated groups (^*^p < 0.01).

As Hsp60 can mediate both protective as well as pro-apoptotic function^23^,^24^,^25^,^26^,^27^, we next sought to determine the exact role of Hsp60 upregulation in the CFEA mediated apoptotic process. To dissect its pro- or anti-apoptotic role, we first examined the status of apoptosis of breast cancer cells where Hsp60 was being upregulated after CFEA treatment. As permeabilization results in loss of Annexin-V staining, vehicle and treated cells were fixed with 4% paraformaldehyde and co-stained with Annexin-V and Hsp60 antibodies for further analysis under confocal microscope. As shown in Fig. 3a, vehicle treated cells have dispersed low Hsp60 expression along with almost negative staining for Annexin-V indicating the presence of non apoptotic healthy cells. In contrast, Hsp60 is shown to be markedly enhanced in treated cells (lower panel), which are also positive for Annexin-V staining suggesting that Hsp60 upregulated cells are in the process of early apoptosis. To further validate the pro-apoptoic role of Hsp60, we performed immunofluoresence costaining of anti-apoptotic protein XIAP (green) along with Hsp60 (red) in fixed and permeabilized cells to show how these two proteins are being regulated after treatment. Here, we observed the downregulation of XIAP as well as strong upregulation of Hsp60 expression in CFEA treated cells compared to control cells (Fig. 3b). Moreover, higher magnification of a merged single cell image of control versus treated group (Fig. 3b, inset) convincingly illustrate that these two proteins are reversibly regulated after CFEA treatment, thereby not only implying the pro-apoptotic function of Hsp60 but also revalidating our array and subsequent western blot data. It is noteworthy to mention that due to the difference in fixation and processing conditions, we observed the variation in basal Hsp60 staining in non-permeabilised (Fig. 3a; second panel) versus permeabilised (Fig. 3b; second panel) cells. Next to determine the pro-apoptotic role of Hsp60, we utilized Hsp60 specific siRNA to knockdown its expression and assessed the effect of CFEA on regulating apoptosis in control and siRNA transfected MCF-7 and MDA-MB-231 cells. Particularly in MCF-7 cells, Hsp60 knockdown alone results in augmentation of anti-apoptotic protein XIAP expression suggesting its direct pro-apoptotic role (Fig. 3c). Further, CFEA treatment did not induce Hsp60 expression under knockdown condition compared to control siRNA transfected cells (Fig. 3c,d). To confirm its pro-apoptotic role, we stained CFEA treated control and Hsp60 siRNA transfected cells with cleaved PARP antibody and assessed under fluorescence microscope. Comparative as well as quantitive data analysis of cleaved PARP positive (green) cells in control versus Hsp60 siRNA transfected CFEA treated cells clearly suggest that Hsp60 plays a pro-apoptotic function in CFEA induced cell death (Fig. 3e; upper panel and lower panel) as indicated by the presence of less number of cleaved PARP positive apoptotic cells (green) in Hsp60 siRNA transfected condition compared to control siRNA transfected cells.

### CFEA promotes accumulation of Hsp60 in the ER

**Figure 4:**
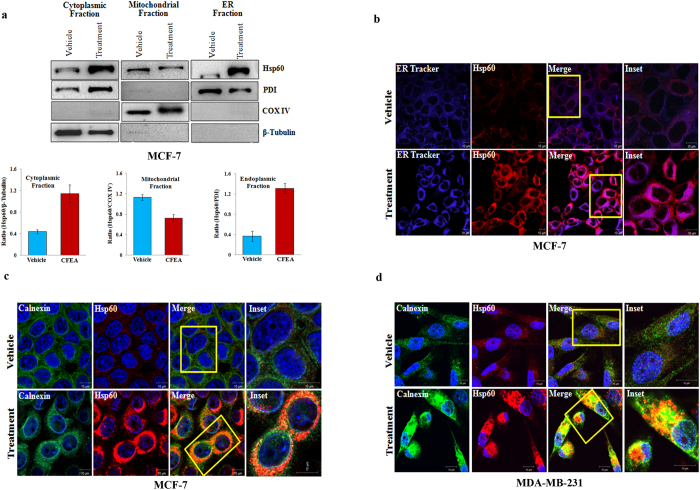
CFEA mediated upregulated Hsp60 is localized in the ER. (**a**) MCF-7 cells treated with either vehicle or CFEA (100 μg/ml) for 24 hours and ER, mitochondria and cytosolic fractions were separated, run on SDS-PAGE and subjected to immunoblot analysis using HSP60, PDI, COX IV, and β-Tubulin antibodies. Bar diagrams (lower panel) show densitometry analysis of Western blot (upper panel) normalized to their respective loading control. (**b–d**), Breast cancer cells were grown in coverslips and treated with either vehicle or CFEA (100 μg/ml) for 24 hours and subjected to immunofluorescence staining and analysed by confocal microscope. Merged confocal photographs represent the superimposition of green and red images and the magnified area of the box were shown in inset pictures. Particularly in panel (**b**), MCF-7 cells were co-stained with Hsp60 (red) antibody and ER tracker (violet) whereas, in panel (**c** and **d**), MCF-7 and MDA-MB-231 cells were co-stained with Hsp60 (red) and Calnexin (green) antibodies. Scale bar, 10 μm. Representative of three independent experiments.

Recent literature suggest that Hsp60 subcellular localization is important in determining the functionality of this protein^19^,^20^,^23^,^30^. Hsp60 is well known to be primarily located in mitochondria but in our previous experiment, we observed that Hsp60 and Annexin-V were co-localized near the outer periphery of the nucleus after CFEA treatment. Annexin-V binds to phosphatidylserine (PS) which is well established to be mainly present in the plasma membrane as well as in ER membrane^31^. This indication prompted us to identify the exact location of Hsp60 upregualtion in breast cancer cells after CFEA treatment. To this endeavor, we first isolated ER, mitochondrial and cytosolic fractions of control and treated MCF-7 cells and analysed for the expression of Hsp60 along with subcellular markers (PDI, COX-IV, and β-Tubulin) by western blot analysis in different fractions. To our great surprise, we found robust upregulation of Hsp60 in the ER fractions of treated cells compared to control (Fig. 4a; upper and lower panels). To confirm the unexpected finding of Hsp60 accumulation in the ER, we co-stained control and treated cells with ER tracker (blue) and Hsp60 (red) and analysed under confocal microscope. In Fig. 4b, Hsp60 was found to be markedly upregulated in treated cells compared to control and upregulated Hsp60 is strongly colocalized with the ER tracker as observed in the merged image of the treated cells. To further validate our observation, we co-stained control and treated (MCF-7 and MDA-MB-231) cells with Hsp60 (red) and ER specific antibody Calnexin (green) and analysed under confocal microscopy. Representative confocal images (Fig. 4c,4d) demonstrate robust Hsp60 upregulation after treatment and clear colocalization of Hsp60 within ER compartments as indicated by the yellow orange merged images (lower bottom inset). Altogether, utilizing multiple approaches, we confirmed that CFEA induced Hsp60 overexpression is localized in the ER compartment and to best of our knowledge this is the first report regarding the accumulation of Hsp60 within the ER during apoptosis.

### Luteolin but not Wedelolactone is the most cytotoxic component of CFEA

**Figure 5:**
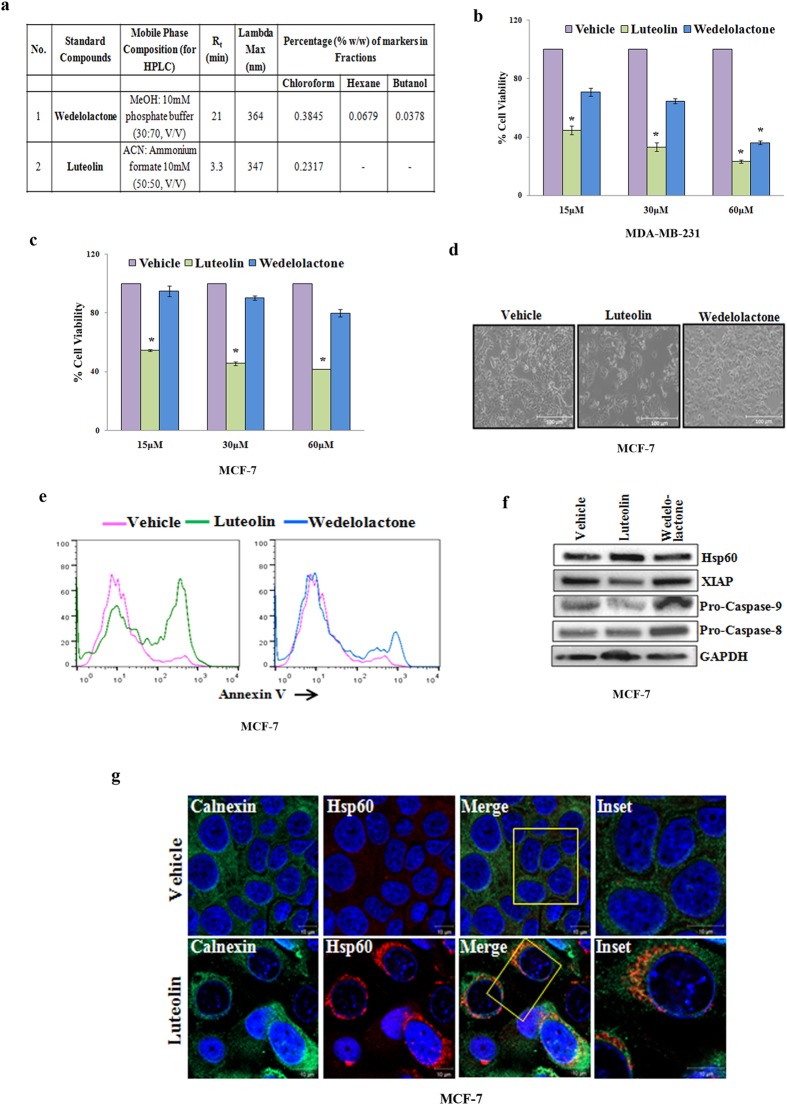
Luteolin but not Wedelolactone is the most cytotoxic component of CFEA. (**a**) Wedelolactone, Luteolin and CFEA at a concentration of 1, 1 and 300 μg/ml respectively were prepared in their mobile phase compositions and their percentage compositions were analyzed on the Shimadzu HPLC system and represented in tabular format. (**b,c**) MCF-7 and MDA-MB-231 cells were treated with increasing concentrations (15-60μM) of wedelolactone (blue bar) and luteolin (green bar) for 24 hours and cytotoxicity were assessed by SRB assay. *Columns*, average of triplicate readings of samples; *error bars*, ± S.D; *p < 0.01; compared to vehicle treated cells. (**d**) Representative photo-micrograph of MCF-7 treated with either vehicle or wedelolactone (60 μM) or luteolin (30 μM) for 24 hours. Scale bar, 100 μm. (**e**) Histogram overlays generated by MCF-7 cells treated with either vehicle or wedelolactone (60 μM) or luteolin (30 μM) for 24 hours, stained with Annexin-V Alexafluor 488 and analysed by flow cytometry. (**f** ) Immunoblot images demonstrate individual western blot analysis of Hsp60, XIAP, Pro-Caspase-8, and Pro-Caspase-9 in either vehicle or wedelolactone (60 μM) or luteolin (30 μM) treated MCF-7 cells for 24 hours. (**g**) MCF-7 cells were grown in coverslips and treated with luteolin (30 μM) for 24 hours and subjected to immunofluorescence staining for Hsp60 and Calnexin and analysed by confocal microscope. Scale bar, 10 μm.

Next, to identify the active components of CFEA, we first performed liquid chromatography-mass spectrometry (LC-MS) of different fractions. As reported in the literature^12^,^13^ our data also suggest that wedelolactone is the major component in almost every fraction of *E. alba*. On the other hand, luteolin was found to be exclusively present in the CFEA pointing towards the fact that it might be the active component responsible for inducing apoptosis in breast cancer cells. To confirm the presence of these two molecules, HPLC was performed. As indicated by LC-MS, HPLC data revealed the exclusive presence of luteolin in CFEA but wedelolactone was found to be ubiquitously present in all fractions. Quantitative analysis determined that the amount of wedelolactone present in CFEA is approximately double the amount of luteolin (Fig. 5a). Therefore, we calculated the amount of both of these constitutents in the effective concentration (100 μg) of CFEA and found out that they were approximately equivalent to 60 and 30 μM of wedelolactone and luteolin respectively. Next, we compared the cytotoxic effect of wedelolactone versus luteolin in MCF-7 and MDA-MB-231 cells by SRB assay. Here, we observed that luteolin is highly cytotoxic to MCF-7 and MDA-MB-231 cells compared to vehicle treated cells. On the other hand, wedelolactone showed very less cytotoxic effect against both the cells particularly in lower (15–30 μM) doses. These results suggest that the presence of luteolin in the CFEA but not wedelolactone is the major component responsible for cytotoxic activity against breast cancer cells (Fig. 5b–d). To further validate the presence of luteolin as most active component for apoptosis, we treated breast cancer cells with luteolin and wedelolactone for 24 hours and assessed the status of Annexin-V staining by flowcytometry. Histogram overlays of treated versus control cells clearly indicate the better efficacy of luteolin over wedelolactone in inducing apoptosis in breast cancer cells (Fig. 5e). To corroborate with CFEA mediated signaling alterations, expression of Hsp60, XIAP, Caspases were assessed in luteolin and wedelolactone treated cells. By western blot analysis, we detected the considerable increase of Hsp60, downregulation of XIAP and pro-caspase-9 but unchanged pro-caspase-8 expression in luteolin treated cells compared to control (Fig. 5f) but not with wedelolactone treatment. Next, we wished to determine the subcellular localization of upregulated Hsp60 after luteolin treatment. Interestingly, similar to CFEA, luteolin treatment also resulted in localization of Hsp60 in the ER as observed by confocal based Hsp60 and Calnexin co-localization studies (Fig. 5g) but not as the similar robustness as it happened in case of CFEA. Altogether, our results suggest that the effective compound of the CFEA is principally luteolin but we can not ignore the possible synergistic effect of diffrent molecules of CFEA.

### CFEA inhibits breast tumor growth *in-vivo* and mitigates tumor induced hepato-renal toxicity

**Figure 6:**
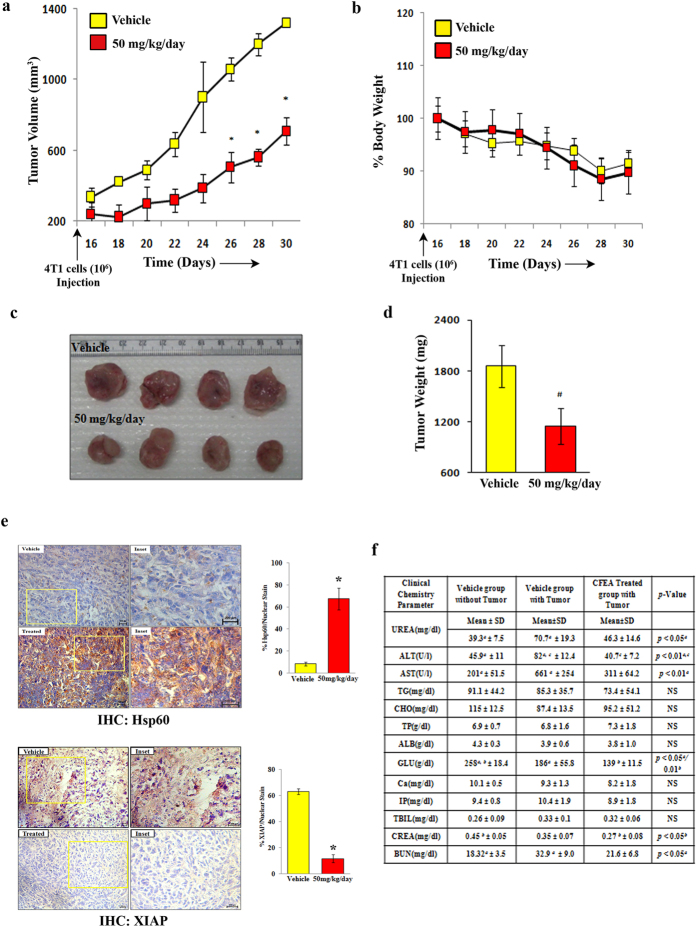
CFEA inhibits breast tumor growth *in-vivo* and mitigates tumor-induced hepato-renal toxicity. 1.0 × 10^6^ mouse breast tumor cells (4T1) were injected subcutaneously into mammary fat pad on right flank of each female Balb/c mouse and when palpable tumors were formed, mice were randomised in two groups (n = 5 in each group) and then fed with either CFEA (50 mg/kg/day) or with vehicle through oral gavage for two weeks. Tumor volume was monitored at regular intervals using an electronic digital caliper. Tumors were harvested 30 days after tumor cell injection. (**a**) Growth curves were shown, where *Points* are indicative of average value of tumor volume; *bars*, +/− SD. The CFEA-treated group had average tumor volumes that were significantly different from the vehicle-treated group (*p < 0.01). (**b**) Average body weight of vehicle and treated mice were represented as line graph. (**c**) Representative tumor images were shown. (**d**) Average tumor weight *bars*, +/− SD of vehicle and treated groups (^#^*p* < 0.05) were displayed in bar diagram. (**e**) Immunohistochemistry were carried out to detect Hsp60 and XIAP in formalin fixed paraffin-embedded sections of both vehicle and CFEA treated mouse breast tumor tissues using anti-Hsp60 (1:100) and anti-XIAP (1:50) antibodies. Representative photomicrographs were shown in top (Hsp60) and bottom (XIAP) panels. Scale bar, 200 μm. Percentage of DAB/Nuclear staining was shown using bar graph (right panels) of respective images. (**f** ) Blood samples were collected before sacrificing the mice and analysed for different biochemical parameters to assess hepato-renal toxicity. ‘*a’* denotes comparison between Vehicle without tumor versus Vehicle with tumor; ‘*b’* signifies Vehicle without tumor versus Treated with tumor. ‘*c’* indicates Vehicle with tumor versus Treated with tumor. ‘NS’ stands for no significant statistical difference between two test groups as well as with their respective controls. *Abbreviations:* alanine aminotransferase (ALT), aspartate aminotransferase (AST), alkaline phosphatase (ALP), triglycerides (TG), total cholesterol (TCHO), total protein (TP), albumin (ALB), Glucose (GLU), calcium (Ca), inorganic phosphorus (IP), total bilirubin (TBIL), creatinine (CREA) and Blood urea nitrogen (BUN). Hepatotoxicity Markers- ALT, AST, ALP, and TBIL; Nephrotoxicity Markers- CREA and BUN.

To evaluate whether the robust cytotoxic effect of CFEA observed *in-vitro* would also result in tumor volume reduction *in-vivo*, we examined the efficacy of CFEA in a syngenic breast tumor mouse model, previously established in our lab^32^. We have selected 50 mg/kg of body weight oral dose of CFEA for our *in-vivo* studies by performing initial pharmacokinetic studies of its active components. Luteolin was found to be detected in mouse blood after 50 mg/kg of body weight oral dose of CFEA (data not shown). 4T1 cells were subcutaneously implanted into the mammary fat pad of Balb/c mice and after the formation of palpable tumors, mice were fed Gum Acacia suspended CFEA or vehicle (Gum Acacia alone) by oral gavage every day for two weeks. Oral administration of CFEA resulted in significant (p ≤ 0.05) reduction of tumor volume, size and weight compared to control fed mice (Fig. 6a,c,d). Moreover, the CFEA was apparently non-toxic to the animals as we did not observe any significant weight loss during the period of treatment (Fig. 6b). To validate our major *in-vitro* finding of Hsp60 upregulation and concomitant XIAP downregulation following CFEA treatment, we performed immunohistochemistry (IHC) to determine the level of these two proteins in harvested control and CFEA treated tumor tissues. As observed in left and right panels of Fig. 6e, CFEA markedly upregulated Hsp60 and reduced XIAP expression in tumors of CFEA treated animals compared to tumors of vehicle treated animals. As liver and kidney functionalities are major indicators of toxic side effects of cancer drugs or nutraceuticals, we then assessed some key parameters of hepatic (ALT, AST, ALP) and renal (CREA and BUN) functions in the serum of normal, tumor bearing vehicle treated, tumor bearing CFEA treated mice. Most interestingly, tumor development resulted in the abnormalities of hepato-renal parameters like increased AST and BUN which are quite expected but instead of imparting toxic side effects, surprisingly CFEA ameliorated a lot of tumor induced hepato-renal abnormalities and improved the function of two major organs (Fig. 6f). Altogether, our data suggest that CFEA not only inhibits *in-vivo* breast tumor growth but also protects mice from its tumor induced hepato-renal toxicity.

## Discussion

Modern day cancer drug discovery largely relies on the purification, synthesis, and administration of a single compound having target specific anti-tumor effect^33^. However, multifactorial and versatile tumorigenic processes change during the course of tumor progression and evidently becomes resistant to that particular drug^34^,^35^. Moreover, target specificity of a single molecule is mostly not unique to cancer cells resulting in severe toxicity to the normal counterpart. Therefore, detailed molecular component analysis and their mechanistic insight of plant extract/fraction having potent anti-tumor effect *in-vitro* and *in-vivo* are powerful tools to find out the most effective combination to selectively target tumor cells in numerous ways to overcome drug resistance and mitigate the toxic side effects. Interestingly, at least in our *in-vitro* cytotoxicity assay, we observed that CFEA posesses significant cytotoxic effect in breast cancer cells but its toxicity is limited to non-tumorigenic breast epithelial cells, whereas, standard FDA approved drugs like doxorubicin, paclitaxel, 5-Fluorouracil were found to have no such selectivity (Fig. 1c). Our unbiased mechanistic search to dissect the apoptotic process revealed the selective involvement of the intrinsic pathway, which is quite distinctive as it contains a mixture of compounds, suggesting that a selective mode of action can also be achievable with naturally ocurring biocombinations. Reprogramming the energy metabolism is one of the central hallmarks^36^ of cancer therapy and activation of intrinsic apoptotic pathways have recently been proposed to be the key, in terms of selective killing of cancer cells by altering its energy homeostasis. As cancer cells have different energy metabolism patterns than normal cells^37^,^38^, tipping these key balances towards cell death machinery by combined molecules could force tumor cells to die without having further scope for the development of treatment resistance.

Precise involvement of Hsp60 in inducing apoptosis in cancer cells over other Hsps by the CFEA clearly indicates the selectivity in the mode of action of this particular fraction. Hsps are well known for cellular protection but Hsp60 has been reported to play a dual role in regulating apoptosis in cells depending on the cell type and context^19^,^20^,^21^,^22^,^23^,^24^,^25^,^26^,^27^. Hsp60 is primarily known to be located in mitochondria^39^ and upto a certain threshold level of Hsp60, it protects cells from death via neutralising cellular stress but its realease or transport to other location may result in apoptosis. In case of tumor cells that have already high Hsp60 level^20^,^21^, further upregulation or its accumulation in different organelles may cause apoptotic catastrophe by activating downstream caspases and inactivating anti-apoptotic proteins.

CFEA mediated cytotoxic effect is aparently selective for cancer cells. This selective cancer cell specific effect can be explained by the difference at the initial level of Hsp60 between normal versus cancer cells. As cancer cells have high basal Hsp60 level compared to normal cells^20^,^21^ and exceeding Hsp60 threshold for tipping the balance towards apoptosis by some agents would be easier for cancer cells than normal cells. Moreover, studies from literature as well as our own observation indicate that not only the regulation of Hsp60 protein expression but also its subcellular localization is the key for its functionality^23^,^26^. Here, we discovered that Hsp60 is localized in the ER of breast cancer cells during apoptosis. ER specific Hsp60 localization directly correlates with apoptosis either by positive Annexin-V staining or by downregulation of anti-apoptotic protein XIAP in the same cells (Fig. 3a,b). The exact role of Hsp60 localization in the ER for causing cell death is not clear yet but earlier Kim *et al.* showed that Hsp60 can be β-O-GlyNAcylated and induces pancreatic β-cell death by releasing Bax, which translocates to mitochondria, also triggering cytochrome *c* release and activates caspase-3^40^. It has been also shown that in fibrosarcoma tumor cells, N-glycosylated Hsp60 is localized in the ER during its release from the cell but their functional significance has not been yet documented^41^. An elegant study by Chandra *et al.* has also shown that cytosolic accumulation of Hsp60 through mitochondrial release is pro-apoptotic in prostate cancer cells^23^. Our LC-MS approach as well as further detailed biological validation indicate that luteolin may be a major component of anti-tumor activity though wedelolactone is also present in good quantity within the same fraction. Previous studies have shown that luteolin is a strong anti-proliferative agent which elicits its effect through multiple mechanisms including downregulation of XIAP^42^,^43^ but its role in regulating Hsp60 during apoptosis is not known so far. Although luteolin was found to be one of the major componets of CFEA it is notworthy to mention that the robustness of effect of CFEA in terms of inducing selective *in-vitro* cytotoxicity along with observed molecular changes are not at per with luteolin driven changes suggesting the contribution of other components of CFEA to have synergistic anti-tumor effect.

In conclusion, we revealed a novel mechanism by which this particular chloroform fraction of *E. alba* inhibits growth of breast cancer cells *in-vitro* and *in-vivo*, and involves specific activation of mitochondrial apoptotic pathways and localization of robustly upregulated Hsp60 in the ER. To the best of our knowledge, this is the first evidence of ER specific localization of Hsp60 during cancer cell apoptosis. From the therapeutic point of view, the anti-cancer effect of this fraction is quite unique as it is not only cytotoxic to cancer cells sparing normal cells, but also ameliorates tumor-induced hepato-renal toxicity *in-vivo*. In addition, our mass fingerprinting studies have identified luteolin as the major effective component of this particular fraction. Finally, our novel observation regarding ER specific Hsp60 localization during apoptosis may potentially open up new avenues for further translational research.

## Methods

### Reagents and antibodies

Luteolin, Wedelolactone, DAPI, Hoechst 33342, JC-1, Doxorubicin, poly-L-lysine solution, Meyer’s Hematoxylin solution, DPX mountant for histology and β-actin were obtained from Sigma Aldrich. Control peptide, pan-caspase inhibitor and caspase-9 inhibitor were purchased from Calbiochem. Dharma-FECT transfection reagent and Hsp60 siRNA were purchased from Dharmacon. Fluorochrome conjugated secondary antibodies, Annexin-V Alexa Fluor 488 and ER-Tracker™ Blue-White DPX were procured from Molecular Probes-Invitrogen. ImmEdge pen (hydrophobic barrier pen), Bloxall blocking solution, DAB peroxidase substrate kit, Vectastain ABC kit were purchased from Vector Laboratories, Inc. Burlingame. Anti-human mouse Hsp60, GAPDH, and β-tubulin antibodies were purchased from Thermo-Fisher whereas, anti-human rabbit Hsp60, Cleaved PARP, Caspase-8, Caspase-9, COX-IV, PDI and Calnexin were obtained from Cell Signaling Technology, Inc. XIAP, PCNA, and HRP-conjugated secondary antibodies were purchased from Santa Cruz Biotechnology. All chemicals and antibodies were obtained from Sigma unless specified otherwise.

### Preparation of plant extract and fractions

Plant material of *E. alba* (L.) was procured and authenticated (voucher No. KRA/24475). Dried grinded plant material (whole plant) was soaked in methanol for 24 hours at RT and percolated four times. Methanol extract was concentrated under reduced pressure using rotary evaporator at 40 °C and fractionated in hexane, chloroform, n-butanol and water. Concentrated dried fractions were stored at 4 °C until use. All fractions were dissolved in cell culture grade DMSO (Sigma) at 200 mg/ml stock.

### Cell culture

Human breast adenocarcinoma MCF-7 and MDA-MB-231, mouse breast cancer 4T1, and non-tumorigenic human breast epithelial cells MCF 10A were obtained from the American Type Culture Collection (ATCC), resuscitated from early passage liquid nitrogen vapour stocks as needed and cultured according to the supplier’s instructions. All experiments were performed within early passages of individual cells.

### Cytotoxicity Assay

A standard colorimetric Sulforhodamine-B (SRB) assay was used for the measurement of cell viability as described before^44^,^45^. The cytotoxic effects of the fractions were calculated as per the formula [100-(Absorbance of treated cells/ Absorbance of vehicle treated cells)] X 100.

### Determination of apoptosis by Flow-cytometry

Induction of apoptosis in human breast cancer cells MCF-7 and MDA-MB-231 was quantitatively determined by flow cytometry using the Annexin-V Alexafluor 488 staining following the manufacturer’s instructions^28^. The stained cells were analyzed by FACS Calibur (Becton Dickinson, USA) and data were analysed by FlowJo software (Tree Star Inc, USA).

### Detection of mitochondrial membrane potential (MMP, ψΔ~m)

The changes in the mitochondrial potential were detected by JC-1, a cationic dye that exhibits potential dependent accumulation in mitochondria, indicated by fluorescence emission shift from red (590 nm) to green (525 nm)^46^. In brief, control and treated breast cancer cells were stained with JC-1 and analyzed by FACS.

### Apoptosis antibody array analysis

MCF-7 cells and MDA-MB-231 cells were treated with either vehicle or CFEA and the apoptosis array analysis was performed using the Proteome Profiler Human Apoptosis Array Kit (ARY009) from R&D Systems according to the manufacturer’s instructions^28^. Array images were analyzed using the ImageJ software (NIH).

### Preparation of subcellular fractions

Mitochondrial and cytoplasmic fractions were isolated from breast cancer cells as per protocol described by Wieckowski *et.al.*^47^ with minor modifications. ER fraction was separated by utilising ER isolation kit (Sigma Aldrich) following manufacturer’s protocol, whereas nuclear extraction was done by utilizing EpiQuik Nuclear Extraction Kit (Epigentek) as per manufacturer’s protocol.

### Western blot analysis

Protein samples were run on 4–15% gradient SDS-polyacrylamide gel (BioRad) and transferred to a PVDF membrane (Millipore, USA)^48^. The membranes were incubated with different primary antibodies and subsequently incubated with peroxidase-linked appropriate secondary antibody. The protein expression was visualized by an enhanced chemiluminescence solution (Immobilon^TM^ western, Millipore, USA) and scanned by gel documentation system (Bio-Rad chemidoc XRS plus). Bands were quantified by densitometry using Image Lab Software (Bio-Rad, USA).

### siRNA knock down experiments

MCF-7 cells were seeded on a 6 well cell culture plate and allowed to grow up to 50% confluent monolayer of cells followed by addition of 50 nM of Hsp60 targeting siRNA or non-targeting control siRNA along with 5 μl of Dharma-FECT transfection reagent in antibiotic free medium as per manufacturer’s protocol. After 48 hours, cells were harvested for protein extraction and estimation followed by Hsp60 knockdown validation using western blot analysis.

### Confocal microscopy

Control and treated cells were fixed with 4% paraformaldehyde in PBS for 10 min at RT and permeabilized by 0.1% NP-40 followed by blocking with 2% BSA for 1 hour at RT. After overnight primary antibody incubation, washed cells were then incubated with fluorescent-conjugated secondary antibodies at RT for 1 hour, followed by DAPI staining for 5 min at RT. After washing, cells were mounted with anti-fade mounting medium on glass slides and viewed under an inverted confocal laser scanning microscope (Ziess Meta 510 LSM; Carl Zeiss, Jena, Germany). Plan Apochromat 63X/1.4 NA Oil DIC objective lens was used for imaging and data collection. Appropriate excitation lines, excitation and emission filters were used for imaging.

### Immunohistochemistry

All tissue specimens were fixed in neutral buffered formalin and embedded in paraffin. For tissue staining, sections were deparaffinised, rehydrated in water, and quenched for endogenous peroxidase. Antigen retrieval was performed in 10 mM sodium citrate buffer (pH 6) for 30 minutes. Processed slides were rinsed in PBS, then the endogenous peroxide activity was neutralized by incubating the slides with bloxall, (blocking solution) for 25 minutes. After blocking, tissue sections were incubated overnight at 4°C with primary antibody against Hsp60 (1:100) and XIAP(1:50), then rinsed with PBS, and incubated with biotinylated secondary antibody for 1 hour at RT, followed by washing with PBS and incubation with ABC reagent (Vector Laboratories) for 1 hour at RT. Slides were incubated with 3′-3′-diamino-benzidine (DAB) as a chromogen and counterstained with haematoxylin. Negative control sections were processed as above without primary antibody incubation. Finally, the sections were dehydrated, cleared, and mounted using DPX. Stained sections were examined under a microscope (Lieca, Germany) under 40x magnification. Immunoratio web application was used for scoring Hsp60 and XIAP staining^49^.

### *In-vivo* tumor development

All animal studies were conducted in accordance with the principles and standard procedures approved by the Institutional Animal Ethics Committee (IAEC) CSIR-Central Drug Research Institute. We have used 4T1 syngenic mouse model to test the *in-vivo* efficacy of CFEA as it better mimics human breast cancer in an immune-competent condition. As described previously^32^, mouse breast cancer 4T1 cells (10^6^) were injected subcutaneously into mammary fat pad on right flank of each 4–6 week old female Balb/c mouse and were allowed to grow palpable tumors. Treatments were given once every day by oral gavage for two weeks. CFEA, suspended 2% Gum Acacia (Gum arabic from acacia tree, purchased from Sigma) in water was used for oral gavage in mice, whereas, Gum Acacia solution alone was used as vehicle for feeding of control group mice. Tumor size was measured using a electronic digital caliper at regular intervals. The volume was estimated by standard formula *V* = *Π*/*6* × *a*^2^ × *b*, wherein *a* is the short and *b* is the long tumor axis^50^. Mice were sacrificed at the end or if complications occurred, which included signs of inactivity, cachexia, or decreased responsiveness.

### Biochemical analysis of blood for hepato-renal factors

Blood samples were collected for serum chemistry analysis in tubes lacking anticoagulant and placed at RT for at least 90 min prior to centrifugation at 1600 g for 10 min. Levels of different hepato-renal factors in the blood of mice were measured using fully automated random access clinical chemistry analyser (Beckman Synchron CX5, USA).

### Mass and HPLC Finger printing

Mass spectrometric detection was performed on API 4000 Q TRAP mass spectrometer (AB Sciex Toronto, Canada) equipped with an electro spray ionization (ESI) source. For HPLC, a standard sample of marker compounds like Wedelolactone, Luteolin and CFEA at a concentration of 1, 1 and 300 μg/ml respectively were analyzed on the Shimadzu HPLC system (Kyoto, Japan) equipped with a LC-20 AD pump, DGU-20A degasser, SIL-HTC Auto sampler, CTO-20AC column oven and a SPD-M20 photo diode array detector. Samples were prepared in their respective mobile phase compositions and their percentage compositions were determined. Chromatographic separation was performed on Phenomenex Luna RP C-18 column (4.6 × 75 mm, 3.0 μm) with the mobile phase flow rate of 1 ml/min. By using LC solution software, chromatograms, retention time (R_t_) and absorbance maxima (nm) were determined.

### Statistical analysis

All *in-vitro* study results shown are representative of at least three independent experiments. Statistical evaluation for *in-vitro* data analysis was determined by Student’s *t* test and two tailed distribution whereas, one way ANOVA was applied to determine statistical significance for *in-vivo* experiments. Differences with *p* ≤ 0.05 were considered statistically significant.

## Additional Information

**How to cite this article**: Arya, R. K. *et al.* Anti-breast tumor activity of Eclipta extract *in-vitro* and *in-vivo*: novel evidence of endoplasmic reticulum specific localization of Hsp60 during apoptosis. *Sci. Rep.* **5**, 18457; doi: 10.1038/srep18457 (2015).

## Supplementary Material

Supporting Information
