# Supplementary material for: Anti-breast tumor activity of Eclipta extract in-vitro and in-vivo: novel evidence of endoplasmic reticulum specific localization of Hsp60 during apoptosis
Source: Sci Rep. 2015 Dec 17;5:18457. doi: 10.1038/srep18457 (PMC4682077; doi:10.1038/srep18457)
Supplement: Supporting Information [file srep18457-s1.pdf]

Anti-breast tumor activity of Eclipta extract *in-vitro* and *in-vivo*: novel evidence of endoplasmic reticulum specific localization of Hsp60 during apoptosis

Rakesh K Arya, Akhilesh Singh, Navneet K Yadav, Srikanth H Cheruvu, Zakir Hossain, Sanjeev Meena, Shrankhla Maheshwari, Anup K Singh, Uzma Shahab, Chetan Sharma, Kavita Singh, Tadigoppula Narender, Kalyan Mitra, Kamal R Arya, Rama K Singh, Jiaur R Gayen, Dipak Datta

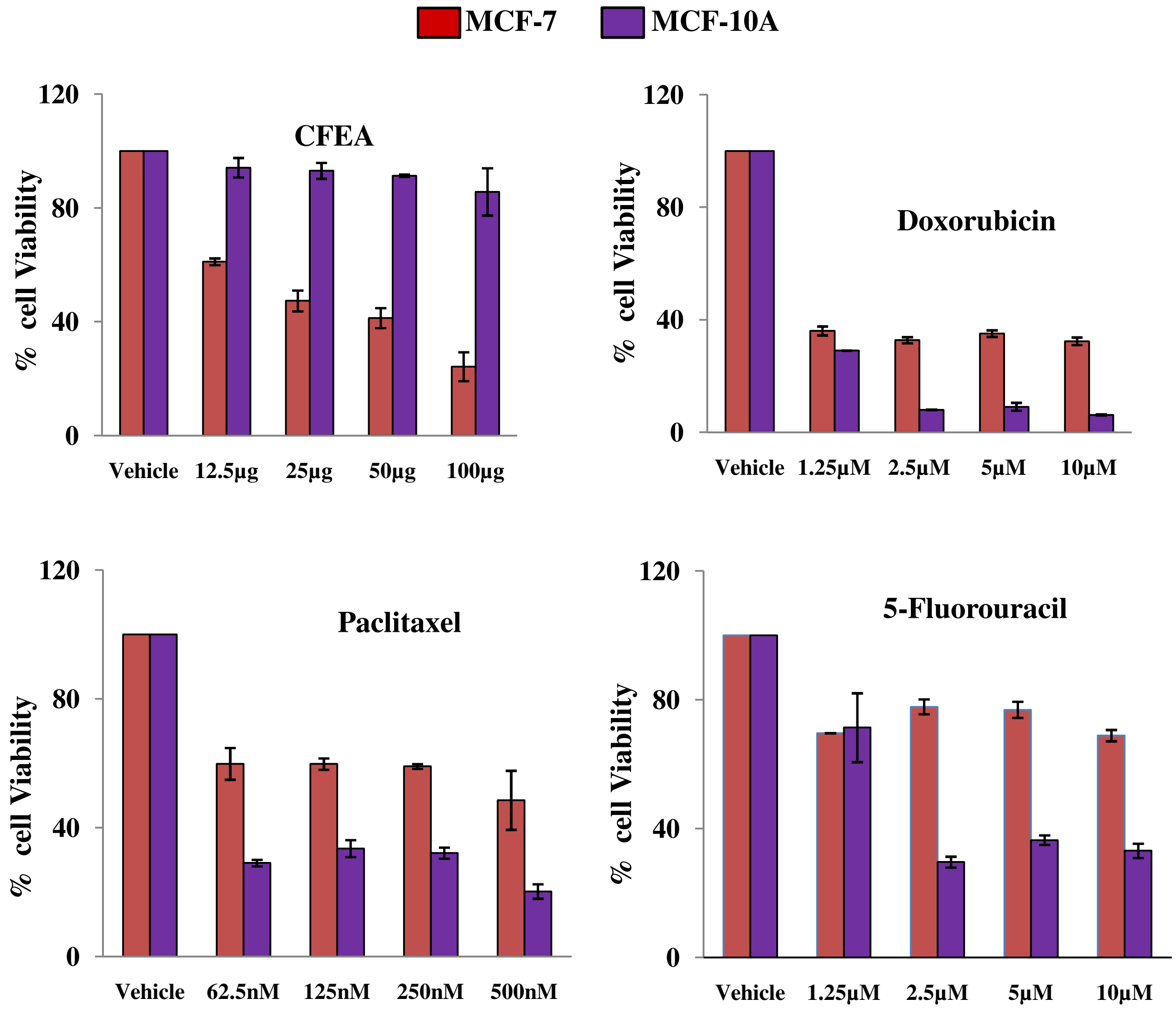

Supplementary Information 1. ***CFEA selectively inhibits breast tumor cell growth*** MCF-7 (red bar) and MCF-10A (violet bar) cells were treated with different doses of CFEA and FDA approved standard anticancer drugs Doxorubicin, Paclitaxel, and 5-Fluorouracil for 48 hours and percent cell viability was assessed by SRB assay. *Columns*, average of quadriplate readings of samples; *error bars*, ± S.D.

Supplemental Information

Coordinate	Target/Control	Coordinate	Target/Control
A1, A2	Positive Control	C13, C14	HO-2
A23, A24	Positive Control	C15, C16	HSP27
B1, B2	Bad	C17, C18	HSP60
B3, B4	Bax	C19, C20	HSP70
B5, B6	Bcl-2	C21, C22	HTRA2/Omi
B7, B8	Bcl-x	C23, C24	Livin
B9, B10	Pro-Caspase-3	D1, D2	PON2
B11, B12	Cleaved Caspase-3	D3, D4	p21/CDNK1A
B13, B14	Catalase	D5, D6	p27/Kip1
B15, B16	cIAP-1	D7, D8	Phoshpo-p53(S15)
B17, B18	cIAP-2	D9, D10	Phoshpo-p53(S46)
B19, B20	Claspin	D11, D12	Phoshpo-p53(S392)
B21, B22	Clusterin	D13, D14	Phoshpo-Rad17
B23, B24	Cytochrome c	D15, D16	SMAC/Diablo
C1, C2	TRAIL R1/DR4	D17, D18	Survivin
C3, C4	TRAIL R2/DR5	D19, D20	TNFR1/TNFRSF1A
C5, C6	FADD	D21, D22	XIAP
C7, C8	Fas/TNFSF6	D23, D24	PBS (-ve Control)
C9, C10	HIF-1α	E1, E2	Positive Control)
C11, C12	HO-1		

Supplementary Information 2. Proteome profiler apoptotic array spot coordinates for target proteins in duplicates were enlisted.

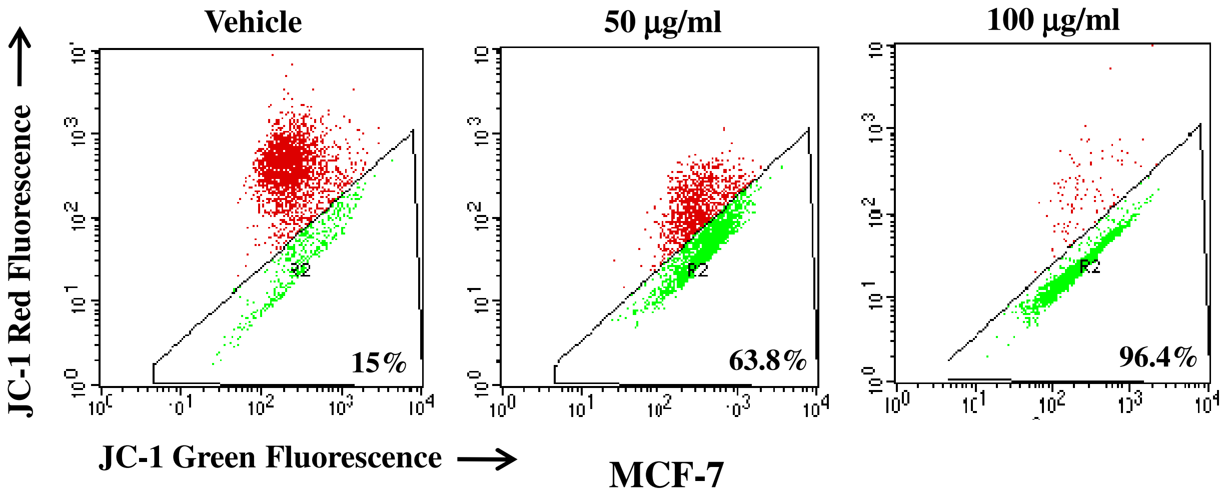

Supplementary Information 3. MCF-7 cells treated with either vehicle or CFEA at different concentrations for 24 hours, were stained with JC-1 dye and the loss of mitochondrial membrane potential was assessed by flow cytometry. Representative of three independent experiments.
